# *chem16S*: community-level chemical metrics for exploring genomic adaptation to environments

**DOI:** 10.1093/bioinformatics/btad564

**Published:** 2023-09-09

**Authors:** Jeffrey M Dick, Xun Kang

**Affiliations:** Key Laboratory of Metallogenic Prediction of Nonferrous Metals and Geological Environment Monitoring of Ministry of Education, School of Geosciences and Info-Physics, Central South University, Changsha, Hunan 410083, China; Key Laboratory of Metallogenic Prediction of Nonferrous Metals and Geological Environment Monitoring of Ministry of Education, School of Geosciences and Info-Physics, Central South University, Changsha, Hunan 410083, China

## Abstract

**Summary:**

The *chem16S* package combines taxonomic classifications of 16S rRNA gene sequences with amino acid compositions of prokaryotic reference proteomes to generate community reference proteomes. Taxonomic classifications from the RDP Classifier or data objects created by the *phyloseq* R package are supported. Users can calculate and visualize a variety of chemical metrics in order to explore the effects of redox, salinity, and other physicochemical variables on the genomic adaptation of protein sequences at the community level.

**Availability and implementation:**

Development of *chem16S* is hosted at https://github.com/jedick/chem16S. Version 1.0.0 is freely available from the Comprehensive R Archive Network (CRAN) at https://cran.r-project.org/package=chem16S.

## 1 Introduction

Chemical analysis of protein sequences can reveal new aspects of genomic adaptation. Carbon oxidation state (*Z*${}_{C}$) represents the degree of oxidation of carbon atoms that results from bonds with other atoms (i.e. H, N, O, and S in the primary sequences of proteins). Protein *Z*${}_{C}$ tends to be lower in the genomes of methanogens that inhabit anoxic environments compared to those that are occasionally found in oxygenated environments (Dick *et al.* 2023). This suggests that geochemical relative stability models are applicable to genomic variation, but identifying the role of the environment is complicated by the cosmopolitan nature of many organisms.

Microbial communities represent a localized collection of genomes. Recently we described the combination of taxonomic abundances from the Ribosomal Database Project (RDP) Classifier (Wang *et al.* 2007) with reference proteomes derived from the Reference Sequence (RefSeq) database of the National Center for Biotechnology Information (NCBI) (O’Leary *et al.* 2016) to generate community reference proteomes (Dick and Tan 2023). This allows new tests of hypotheses about genomic adaptation. For instance, the thermodynamic prediction that protein *Z*${}_{C}$ is positively correlated with environmental oxidation–reduction potential has been confirmed for bacterial communities at a global scale (Dick and Meng 2023).

Community reference proteomes are inferred by taxonomic comparisons with genomic databases rather than directly derived from protein extracts or community DNA (i.e. metaproteomes and metagenomes). Despite this, trends of *Z*${}_{C}$ for community reference proteomes are mostly consistent with those for protein sequences inferred from shotgun metagenomes (Dick and Tan 2023). Different natural abundances and extraction efficiencies for cytoplasmic and membrane proteins in metaproteomic experiments can explain in part the relatively weak correspondence of *Z*${}_{C}$ between metaproteomes and community reference proteomes (Dick and Meng 2023). For these reasons, metrics calculated for community reference proteomes are indicators of genomic variation rather than protein expression.

The code that was developed in our recent studies was consolidated to create the *chem16S* package. An important new addition to the package are reference proteomes derived from the Genome Taxonomy Database (GTDB) (Parks *et al.* 2022). Using the GTDB for both taxonomic classification and reference proteomes avoids the uncertain mapping between the RDP training set and the NCBI taxonomy. Moreover, functions have been added to *chem16S* to process data objects generated with the *phyloseq* package (McMurdie and Holmes 2013), allowing for seamless calculation and visualization of chemical metrics for microbial communities by more researchers.

## 2 Implementation

*chem16S* is written in R (R Core Team 2023) and is structured around three core functions. *read_RDP* reads taxonomic classifications from an output file of the RDP Classifier, removes domain-level and certain nonprokaryotic classifications (e.g. Chloroplast), then retains the lowest-level classification for each input sequence from genus to phylum level. *map_taxa* maps classifications to the NCBI taxonomy by automatic matching of both taxonomic rank and name together with manual mapping for some taxa (see Dick and Tan 2023); an option is available to modify the manual mappings at runtime. Then, *get_metrics* combines the abundances of mapped taxa with precomputed amino acid compositions of reference proteomes for taxa in order to obtain the community reference proteome for each sample.

Users can select from a variety of chemical metrics, including the aforementioned *Z*${}_{C}$. A projection from elemental composition to chemical composition by means of thermodynamic components (aka basis species) permits the calculation of stoichiometric oxidation state (*n*${}_{O_{2}}$) and hydration state (*n*${}_{H_{2}O}$). For protein sequences in a given genome, the element-based metric *Z*${}_{C}$ and reaction-based metric *n*${}_{O_{2}}$ strongly covary, but *n*${}_{H_{2}O}$ is largely decoupled from *Z*${}_{C}$ (Dick 2022); salinity is one factor that may modulate the differences of *n*${}_{H_{2}O}$ between free-living communities (Dick *et al.* 2020). Other metrics available in *chem16S* are elemental ratios (H/C, N/C, O/C, and S/C), basic physicochemical quantities including protein length and molecular weight of amino acid residues, and derived quantities including grand average of hydropathicity (GRAVY) and isoelectric point (pI); the latter two are modeled after the ProtParam tool in UniProt (Gasteiger *et al.* 2005).

RefSeq-based reference proteomes and manual mapping between RDP and NCBI taxonomies were described previously (Dick and Tan 2023). However, inconsistencies between these taxonomies preclude a completely accurate mapping. For this reason, a new set of reference proteomes was generated from the Genome Taxonomy Database (GTDB release 207) using a script that is available in the package. Taxonomic assignments of 16S rRNA gene sequences made using a separately maintained GTDB training set (Alishum 2022) formatted for the DADA2 sequence processing package (Callahan *et al.* 2016) in principle ensure complete mapping to the GTDB reference proteomes, and a 100% mapping rate has been observed for analyses performed in this study (see below).

The *phyloseq* package supports analysis and visualization of microbiome data (McMurdie and Holmes 2013). *chem16S* can analyze taxonomic abundances in the OTU table of a “phyloseq-class” object; this table may hold either operational taxonomic units or amplicon sequence variants (ASVs), depending on the sequence processing pipeline used. Functions are provided to generate a table of lowest-level taxonomic classifications (*ps_taxacounts*), calculate chemical metrics for community reference proteomes (*ps_metrics*), and plot either individual metrics as a function of sample data (*plot_ps_metrics*; facets are used to visualize trends of multiple metrics) or two chemical metrics against each other (*plot_ps_metrics2*) using ggplot2 graphics (Wickham 2016). The plotting functions integrate the lower-level functions, so a single function call runs the calculations and makes a plot.

## 3 Applications

**Figure 1.**
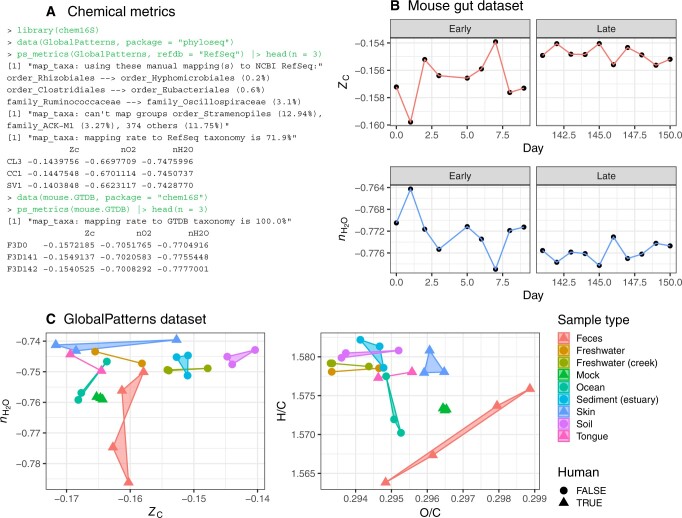
(A) Code example showing calculation of chemical metrics for two datasets. The most abundant unmapped taxon in the GlobalPatterns dataset (Stramenopiles) is a eukaryotic order, so no mapping to archaeal or bacterial taxa is possible. (B) Individual chemical metrics for the mouse gut dataset plotted against days postweaning. (C) Pairs of chemical metrics for the GlobalPatterns dataset.

Two short examples illustrate the benefits of using *chem16S* to represent communities in terms of chemically interpretable variables. The mouse.GTDB “phyloseq-class” object was produced in this study by following the DADA2 Pipeline Tutorial version 1.16 (https://benjjneb.github.io/dada2/tutorial.html, accessed on 7 July 2023) modified to use the GTDB training set for taxonomic classification. The source data were downloaded from https://mothur.s3.us-east-2.amazonaws.com/wiki/miseqsopdata.zip (accessed on 7 July 2023) and represent a progression of gut microbiome composition in a single mouse from early (0–9 days) to late (141–150 days) times postweaning (Schloss *et al.* 2012). There is 100% mapping of taxonomic classifications to the GTDB-based reference proteomes, and the chemical analysis reveals anticorrelated trends in *Z*${}_{C}$ and *n*${}_{H_{2}O}$ between early and late samples (Fig. 1A and B). A progressive decrease of whole-body body water content is a characteristic of mammalian development (Moulton 1923), so the decreasing trend of *n*${}_{H_{2}O}$ that is apparent in the community reference proteomes suggests a physicochemical link to host physiology.

The GlobalPatterns dataset that is part of the *phyloseq* package is based on data from Caporaso *et al.* (2011), in which taxonomy was assigned using the RDP Classifier. After the dataset is loaded, running the *ps_metrics* function displays messages about manual mappings to the NCBI taxonomy and percentages of the most abundant unmapped taxa and returns values for three default chemical metrics (Fig. 1A). The values of *Z*${}_{C}$ delineate relatively reduced (e.g. tongue and ocean) and oxidized (in particular, soil) community reference proteomes and are largely decoupled from *n*${}_{H_{2}O}$ (Fig. 1C). Consistent with previous observations for metagenomes (Dick *et al.* 2020), the range of *n*${}_{H_{2}O}$ for ocean samples is lower than that for freshwater samples. The largest variation of *n*${}_{H_{2}O}$ does not occur between sample types but rather within the group of fecal samples, some of which exhibit extremely low *n*${}_{H_{2}O}$. Unlike other sample types, fecal samples are characterized by a tight linear covariation between H/C and O/C with a slope that is somewhat >2, which likewise suggests that H_2_O is an influential thermodynamic component in the genomic variability among fecal communities.

The package includes three vignettes—accessible online at CRAN and locally after the package installed—that cover patterns of chemical metrics in the reference proteomes of prokaryotic organisms and viruses (metrics.Rmd), integration of *chem16S* with *phyloseq* (phyloseq.Rmd), and plots of two chemical metrics for visualizing trends within and between datasets (plotting.Rmd). In the latter vignette, differences of community *Z*${}_{C}$ and *n*${}_{H_{2}O}$ between sediment in the eastern Pacific Ocean and hot springs in the Qinghai-Tibet Plateau (Fonseca *et al.* 2022, Zhang *et al.* 2023) are used to illustrate potential effects of redox conditions and salinity on genomic adaptation at a global scale.

## 4 Conclusions

The merger of taxonomic and genomic information in community reference proteomes allows microbial communities to be represented in chemical terms. *chem16S* makes it easy to use taxonomic classifications produced by the RDP Classifier or OTU/ASV tables in “phyloseq-class” objects to calculate and visualize chemical metrics for community reference proteomes and thereby to gain new insight into the role of physicochemical factors in genomic adaptation to environments.

## Data Availability

The code used to make Fig. 1 is available at https://github.com/jedick/JMDplots/blob/main/R/chem16S.R.

## References

[btad564-B1] Alishum A. DADA2 Formatted 16S rRNA Gene Sequences for Both Bacteria & Archaea. Version 4.3. Zenodo, 2022. 10.5281/zenodo.6655692.

[btad564-B2] Callahan BJ , McMurdie PJ, Rosen MJ et al DADA2: high-resolution sample inference from Illumina amplicon data. Nat Methods 2016;13:581–3. 27214047 10.1038/nmeth.3869 PMC4927377

[btad564-B3] Caporaso JG , Lauber CL, Walters WA et al Global patterns of 16S rRNA diversity at a depth of millions of sequences per sample. Proc Natl Acad Sci USA 2011;108:4516–22. 20534432 10.1073/pnas.1000080107 PMC3063599

[btad564-B4] Dick JM. A thermodynamic model for water activity and redox potential in evolution and development. J Mol Evol 2022;90:182–99. 35279735 10.1007/s00239-022-10051-7

[btad564-B5] Dick JM , Meng D. Community- and genome-based evidence for a shaping influence of redox potential on bacterial protein evolution. mSystems 2023;8:e00014-23. 37289197 10.1128/msystems.00014-23 PMC10308962

[btad564-B6] Dick JM , Tan J. Chemical links between redox conditions and estimated community proteomes from 16S rRNA and reference protein sequences. Microb Ecol 2023;85:1338–55. 35503575 10.1007/s00248-022-01988-9

[btad564-B7] Dick JM , Yu M, Tan J. Uncovering chemical signatures of salinity gradients through compositional analysis of protein sequences. Biogeosciences 2020;17:6145–62.

[btad564-B8] Dick JM , Boyer GM, Canovas PA et al Using thermodynamics to obtain geochemical information from genomes. Geobiology 2023;21:262–73. 36376996 10.1111/gbi.12532

[btad564-B9] Fonseca A , Espinoza C, Nielsen LP et al Bacterial community of sediments under the Eastern Boundary Current System shows high microdiversity and a latitudinal spatial pattern. Front Microbiol 2022;13:1016418. 36246233 10.3389/fmicb.2022.1016418 PMC9561620

[btad564-B10] Gasteiger E , Hoogland C, Gattiker A et al Protein identification and analysis tools on the ExPASy server. In: Walker JM (ed.), The Proteomics Protocols Handbook. Totowa, NJ: Humana Press, 2005, 571–607.

[btad564-B11] McMurdie PJ , Holmes S. phyloseq: an R package for reproducible interactive analysis and graphics of microbiome census data. PLoS One 2013;8:e61217. 23630581 10.1371/journal.pone.0061217 PMC3632530

[btad564-B12] Moulton CR. Age and chemical development in mammals. J Biol Chem 1923;57:79–97.

[btad564-B13] O'Leary NA , Wright MW, Brister JR et al Reference sequence (RefSeq) database at NCBI: current status, taxonomic expansion, and functional annotation. Nucleic Acids Res 2016;44:D733–45. 26553804 10.1093/nar/gkv1189 PMC4702849

[btad564-B14] Parks DH , Chuvochina M, Rinke C et al GTDB: an ongoing census of bacterial and archaeal diversity through a phylogenetically consistent, rank normalized and complete genome-based taxonomy. Nucleic Acids Res 2022;50:D785–94. 34520557 10.1093/nar/gkab776 PMC8728215

[btad564-B15] R Core Team. R: a Language and Environment for Statistical Computing. Vienna, Austria: R Foundation for Statistical Computing, 2023.

[btad564-B16] Schloss PD , Schubert AM, Zackular JP et al Stabilization of the murine gut microbiome following weaning. Gut Microbes 2012;3:383–93. 22688727 10.4161/gmic.21008 PMC3463496

[btad564-B17] Wang Q , Garrity GM, Tiedje JM et al Naïve Bayesian classifier for rapid assignment of rRNA sequences into the new bacterial taxonomy. Appl Environ Microbiol 2007;73:5261–7. 17586664 10.1128/AEM.00062-07 PMC1950982

[btad564-B18] Wickham H. ggplot2: Elegant Graphics for Data Analysis, 2nd edn. Berlin: Springer, 2016.

[btad564-B19] Zhang H-S , Feng Q-D, Zhang D-Y et al Bacterial community structure in geothermal springs on the Northern edge of Qinghai-Tibet Plateau. Front Microbiol 2023;13:994179. 37180363 10.3389/fmicb.2022.994179 PMC10172933

